# Clinical Utility of the Contrast-Enhanced Endoscopic Ultrasound Guided Fine Needle Aspiration in the Diagnosis of Pancreatic Cyst

**DOI:** 10.3390/diagnostics12092209

**Published:** 2022-09-12

**Authors:** Miruna Patricia Olar, Sorana D. Bolboacă, Cristina Pojoga, Ofelia Moșteanu, Marcel Gheorghiu, Radu Seicean, Ioana Rusu, Zeno Sparchez, Nadim Al Hajjar, Andrada Seicean

**Affiliations:** 1 Department of Gastroenterology, Iuliu Hațieganu University of Medicine and Pharmacy, Croitorilor Str., no. 19-21, 400162 Cluj-Napoca, Romania; 2 Department of Medical Informatics and Biostatistics, Iuliu Hațieganu University of Medicine and Pharmacy, Louis Pasteur Str., no. 6, 400349 Cluj-Napoca, Romania; 3 Regional Institute of Gastroenterology and Hepatology, Croitorilor Str., no. 19-21, 400162 Cluj-Napoca, Romania; 4 Department of Clinical Psychology and Psychotherapy, International Institute for Advanced Study of Psychotherapy and Applied Mental Health, Babeș-Bolyai University, Sindicatelor Str, no. 7, 400029 Cluj-Napoca, Romania; 5 First Department of Surgery, Iuliu Hațieganu University of Medicine and Pharmacy, Clinicilor Str., no. 3-5, 400006 Cluj-Napoca, Romania; 6 Third Department of Surgery, Iuliu Hațieganu University of Medicine and Pharmacy, Croitorilor Str., no. 19-21, 400162 Cluj-Napoca, Romania

**Keywords:** CH-EUS (contrast-enhanced endoscopic ultrasound), EUS-FNA (endoscopic ultrasound fine needle aspiration), endoscopic ultrasound, mural nodule, pancreatic cyst

## Abstract

Endoscopic ultrasound fine needle aspiration (EUS-FNA) cytology from an intracystic fluid is useful in the differentiation of pancreatic cysts, with low sensitivity, which increases when the solid component is targeted. The clinical utility of contrast-enhanced guided EUS-FNA (CH-EUS-FNA) in the solid component is not known. We aimed to assess the diagnostic value of CH-EUS-FNA in enhanced mural nodules and discrimination between different cysts using contrast-enhanced endoscopic ultrasound (CH-EUS). The prospective study recruited patients with pancreatic cysts with an unclear diagnosis. The CH-EUS was followed by CH-EUS-FNA. The final diagnosis was based on surgery or the correlation between clinical history, cross-sectional imaging, echoendoscopic morphology, cystic fluid analysis, and follow-up. Fifty-eight patients with pancreatic cysts were evaluated. The mucinous cysts had wall arterial enhancement more often than non- mucinous cysts (*p* < 0.0001), with 90.2% sensitivity and 70.6% specificity. The CH-EUS-FNA from cystic fluid and mural nodules identified mucinous cysts and malignancy with 82.4% and 84.2% sensitivity and 92% and 100% specificity. Twenty-one cysts had solid components, but only 13 were enhanced mural nodules on EUS assessment with conclusive cytology in all cases and malignancy in 76.9%. Contrast-enhanced endoscopic ultrasound should be completed in all PCN with solid components in order to avoid unnecessary EUS-FNA and to guide FNA for the identification of malignant cysts.

## 1. Introduction

Due to the increasing use of cross-sectional imaging techniques for varied medical conditions, more and more pancreatic cysts are incidentally found. Magnetic resonance imaging (MRI) revealed a prevalence of incidental pancreatic cystic neoplasm (PCN) in adults of 13.5–49.1% [1]. The malignancy rate among PCN varies from 15% to 42% in surgical series, depending on whether all the resected cysts are included or only intrapapillary mucinous ductal neoplasms (IPMN) [2]. The higher malignancy risk was found in main-duct IPMN, being over 62%, meanwhile, in the mucinous cystic neoplasms (MCN), it was below 34% [3]. In the case of asymptomatic side branch-IPMNs, about 3.5% progress to malignancy. The presence of mural nodules is associated with a five-time risk of developing malignancy [4].

The discrimination between the different cysts is crucial for the therapeutic approach. The cyst morphology can be similar and sometimes, it is a challenge to diagnose them precisely. The accuracy of the specific diagnosis of is 40–95% with MRI, 40–81% with computer tomography (CT), and 48–84% with EUS without fine-needle aspiration (FNA) [2,3,4,5]. The contrast-enhanced EUS (CH-EUS) is useful in discriminating the mural nodules from mucus [5] and in differentiating pseudocysts from PCN [6,7,8]. A meta-analysis summarizing the CH-EUS studies proved that the sensitivity and specificity for diagnosing malignancy within mural nodules are 97% and 90%, respectively [9]. 

EUS-FNA cytology from an intracystic fluid is helpful in diagnosing cysts >15 mm with low sensitivity (27–48%) but high specificity (83–100%) [10]. Targeting the solid component of PCN increases the diagnostic yield by 29–37% [11], but its role in conjunction with CH-EUS has not been studied yet.

We aimed to assess the diagnostic value of contrast-guided EUS-FNA (CH-EUS-FNA) in enhanced mural nodules and to discriminate malignant versus non-malignant pancreatic cysts and mucinous versus non-mucinous cysts by using CH-EUS features.

## 2. Materials and Methods

### 2.1. Study Design

This prospective study was conducted between April 2018 and May 2020 at one tertiary medical center, with approval from the institutional review board. All patients gave written informed consent for CH-EUS and EUS-FNA, according to Helsinki guidelines. The study was registered on ClinicalTrials.gov (NCT04389892).

### 2.2. Subjects and Data Collection

Consecutive patients aged 18–90 years diagnosed with an unclear specific diagnosis of PCN on CT or MRI with PCN > 15 mm were recruited for the study. Exclusion criteria were: (1) refusal to participate, or contraindications of proposed intervention; (2) platelet level < 50000/mm^3^ and INR (international normalized ratio) >1.5; (3) patients with a solid mass with <50% cystic component; (4) presence of any of the following: duodenal stenosis, severe chronic pancreatitis, history of pancreatic cancer or major upper abdominal surgery, history of acute pancreatitis within three months; (5) congestive heart failure; (6) known allergy to Sonovue; (7) lack of specific diagnosis after CH-EUS-FNA procedure.

### 2.3. Study Outcome and Definitions

The primary outcome was to establish the diagnostic value of CH-EUS-FNA through the enhanced solid component in PCN. The second outcome was finding specific CH-EUS features in mucinous versus non-mucinous PCN, and in malignant versus non-malignant PCN. 

The patients were analyzed by EUS, and contrast-enhanced qualitative analysis followed by EUS-FNA if the PCN was without a typical diagnosis on EUS and CH-EUS. The solid component was considered the hypoechoic component inside the cysts, with or without vascularity. Mural nodules were considered the solid components, with hypoechoic aspect and vascularity on power Doppler or CH-EUS.

The cytology result of CH-EUS-FNA from the enhanced solid component and from fluid analysis (cytology, carcinoembryonic antigen—CEA) in terms of conclusive results and dysplasia or malignancy were noted. The cytology was considered adequate if the samples were sufficiently cellular [12]. The specimen with unequivocally positive malignant cells or high-grade dysplasia was considered positive for malignant PCN and represented one criterion for surgery [10]. The CEA cut-off value for differentiating between a serous cystadenoma and a mucinous PCN was >192 ng/mL [3].

Mucinous PCNs were considered IPMN, MCN, cystic acinar cell carcinoma, and cystic ductal adenocarcinoma. Non-mucinous PCNs were considered serous cystic neoplasms (SCN) and pseudocysts [10,13,14]. Indeterminate cysts were considered when imaging modality or cyst fluid analysis via EUS was not diagnostic and were excluded from further analysis. The indication for surgery was based on guideline recommendations [10]. 

Follow-up of those patients that fit surgery but without surgical indication at the initial diagnosis consisted of clinical examination, CA 19-9 antigen, MRI, and/or EUS at 12 months. 

The final diagnosis was based on surgical pathology or EUS-FNA cytology or on the correlation of clinical history, CT/MRI, B-mode EUS, and CH-EUS morphology, cyst fluid analysis (cytology from the cyst fluid or the solid component, CEA) and follow-up (clinical evaluation, serum CA19-9, abdominal ultrasound every six months and EUS or MRI every year for patients without surgical indication). 

The arterial enhancement (contrast uptake) was considered the first 25–30 s after injection and the venous phase (wash-out) 30–45 s after injection [15]. For the venous phase, wash-out of Sonovue was classified as fast (between 30–45 s from the contrast injection) or slow (after 45 s from the contrast injection), compared to the normal pancreatic tissue. 

### 2.4. Procedure

All interventions were performed using a therapeutic linear array echoendoscope (GF-UCT 180 AL5; Olympus, Tokyo, Japan) with an Aloka Prosound F75 ultrasound machine equipped with extended pure harmonic detection. All interventions were undertaken by the three authors (A.S., O.M, and C.P.), who were experienced in this type of procedure. Patients were under light sedation (intravenous midazolam) or deep sedation (propofol). The patient was positioned in the left lateral decubitus.

The tracking parameters for B-mode EUS were the cyst morphology (the wall, the septum) and the presence or absence of solid components. Intracystic mucus appears as a smooth, well-defined hyperechoic rim with a hypoechoic center compared with the surrounding parenchyma. True epithelial nodules have ill-defined borders and a hyperechoic center [16].

For CH-EUS assessment, 2.4 mL contrast agent (Sonovue; Bracco, Milan, Italy) was rapidly injected intravenously, followed by 5 mL of flushing saline, according to the European Federation of Societies for Ultrasound in Medicine and Biology recommendation [17]. 

The frequency used was 7.5 MHz and a low mechanical index of 0.20. After a careful examination in the B mode of the entire pancreas, the image was fixed on the region of interest (pancreatic cyst), and the extended pure harmonic detection mode was selected. Each lesion was observed using CH-EUS mode for 120 s.

Cystic wall and nodule vascularization was defined as visible contrast enhancer bubble movement within the cystic wall or nodules. During the arterial phase (25–30 s from injection), the enhancement pattern was defined as follows: nonenhancement, no contrast uptake seen; hypoenhancement, less uptake of contrast than the surrounding parenchyma; and hyperenhancement, uptake in the mass greater than in the surrounding parenchyma. 

The CH-EUS-FNA towards the most enhanced part of the cysts was performed with 19 G or 22 G needles (for pancreatic head cyst) (Expect; Boston Scientific, Marlborough, MA, USA). Only one passage was performed, first, the liquid was aspirated, and then a sample of the hyperenhanced wall or solid component was taken, if present.

The patient was observed for one hour and discharged if uneventfully. After CH- EUS-FNA, the patients started the antibiotic prophylaxis with ciprofloxacin every 12 h for three days, according to the guidelines available when the study started. 

### 2.5. Preparation of Samples

No cytopathologist was present when the samples were collected and no through-needle biopsy was performed. The liquid was analyzed macroscopically for color and viscosity. The sample bottle was sent to the laboratory for CEA and cytology. When a small quantity of liquid was obtained (1 mL), cytology was preferred instead of CEA detection. 

The cytoblock technique for cytology analysis was used in every case with the following considerations: mucin-containing cells for IPMN or MCN; glycogen-containing cells for SCN; inflammatory cells or non- mucinous epithelium for pseudocysts [18]. 

When a core was expelled by re-introduction of the stylet, this was put into 10% buffered formalin. The specimens were embedded in paraffin and stained with hematoxylin-eosin-safran, with or without immunohistochemistry sections.

Specimens were independently analyzed by two pathologists (I.R. and D.R.), who had access to the clinical and imaging information.

### 2.6. Statistical Analysis

The results were expressed as numbers, percentages, or reports for qualitative variables. The patient’s age and body mass index were reported as mean ± SD (standard deviation) since data proved to follow the theoretical normal distribution. The size of the lesions was summarized as median and interquartile range. Chi-squared or Fisher’s exact test were used to test the associations in the contingency tables according to the expected frequencies.

The performances of specific EUS technique were reported relative to the final diagnostic using the most used metrics (Se-sensitivity = TP/(TP + FN); Sp-specificity = TN/(TN + FP); PPV-positive predictive value = TP/(TP + FP); NPV-negative predictive value = TN/(TN + FN); Acc-accuracy = (TP + TN)/(TP + FP + FN + TN);+LR-positive likelihood ratio = Se/(100 − Sp); -LR-negative likelihood ratio = (100 − Se)/Sp, where TP = true positive cases, TN = true negative cases, FP = false positive cases, FN = false negative values). Positive and negative Clinical Utility Index (CUI) was calculated with an Excel program (available at: https://www.psycho-oncology.info/cui.html, accessed on 11 November 2021)) implemented following the formulas introduced by Mitchell [19]. Statistical analysis was conducted at a significance level of 5%, so the *p*-values less than 0.05 were considered statistically significant.

## 3. Results

**Figure 1:**
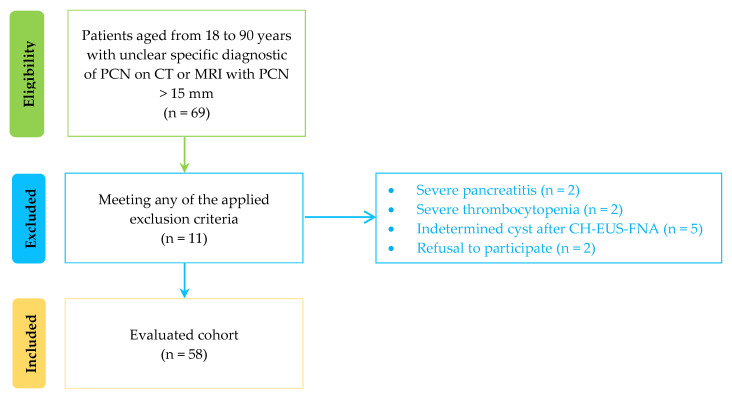
Study population flowchart (PCN- pancreatic cystic neoplasm; CT—computer tomography; MRI- magnetic resonance imaging; CH-EUS-FNA- contrast-enhanced guided endoscopic ultrasound fine needle aspiration).

Sixty-nine subjects were referred for differential diagnosis of a pancreatic cyst during the study period. Eleven patients were excluded, two with severe chronic pancreatitis, two with severe thrombocytopenia, two due to refusal to participate, and five cysts remained indeterminate after the CH-EUS-FNA procedure (Figure 1).

### 3.1. Patient Characteristics

Fifty-eight patients, aged from 23 to 86 years, fulfilled the inclusion criteria (Table 1). The final diagnosis was based on surgery in 17 (29%), on CH-EUS-FNA cytology in eight of malignant cysts (six had contraindications for surgery because of arterial invasion or comorbidities and two patients refused the surgical treatment), or on the combination of CH-EUS features, intracystic fluid analysis and follow-up in 33 patients.

All patients were assessed by CT and 28 (48.27%) by MRI at the time of patient inclusion. The median follow-up was 27.5 months (Q1 to Q3 = 12.0 to 35.0 months), with 14 deaths (13 related to PCN history).

### 3.2. Standard EUS Assessment 

EUS showed a microcystic aspect in eight cases, a micro-macro-cystic aspect in 33 cases, and a macrocystic aspect in 19 cases. The solid component was noted in 21 lesions, but only four (19%) had microvessels on Power Doppler examination, and three (14.3%) had contrast uptake on CT scan (Supplementary Material—Table S1).

### 3.3. CH-EUS in Diagnosing Pancreatic Cysts

**Figure 2:**
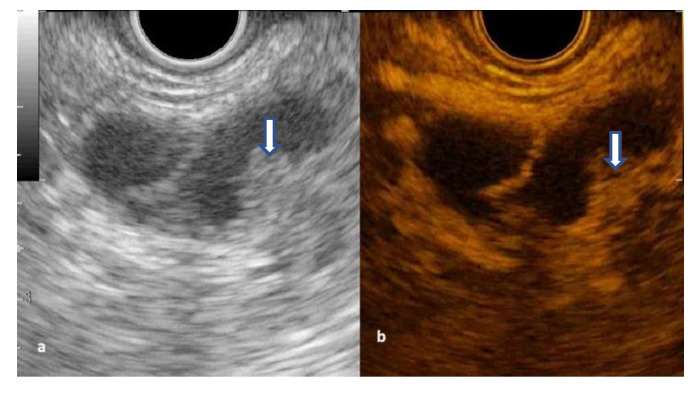
Secondary branch intrapapillary mucinous neoplasms with mural nodule (arrow): (**a**) B-mode EUS; (**b**) Contrast-enhanced EUS.

**Figure 3:**
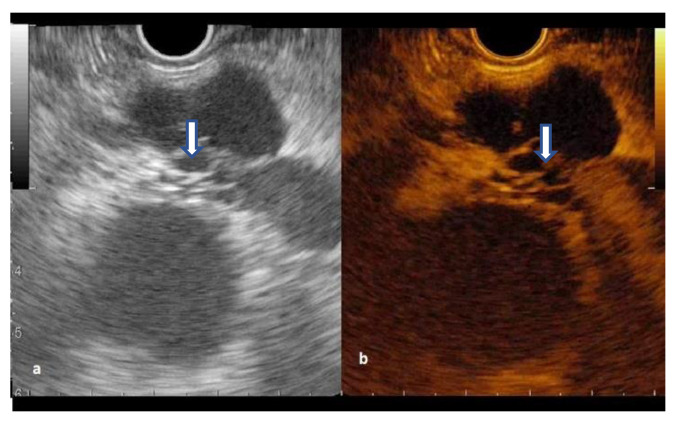
Serous cystic neoplasm with honeycomb appearance (arrow): (**a**) B-mode EUS; (**b**) Contrast-enhanced EUS.

The cystic wall was hyperenhanced in all IPMNs and MCNs, and half of SCNs and cystic ductal adenocarcinoma (Figure 2 and Figure 3). The venous wash-out was fast in 13 (43%) of IPMN and three (75%) of MCN.

**Figure 4:**
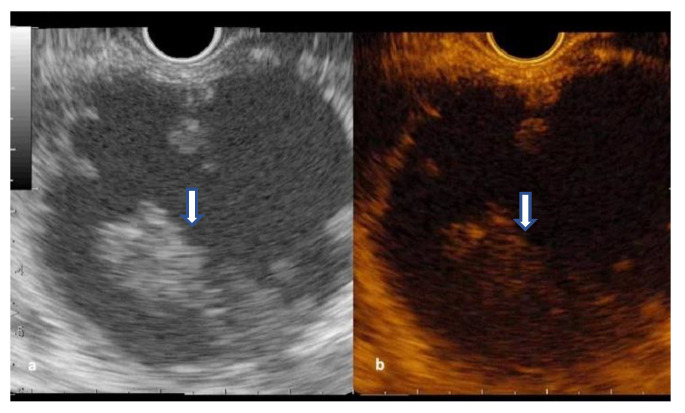
Walled-off pancreatic necrosis with an unenhanced solid component (arrow): (**a**) B-mode EUS; (**b**) Contrast-enhanced EUS.

**Figure 5:**
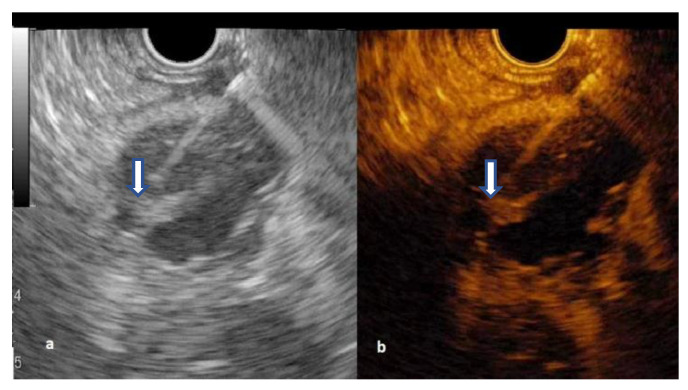
Branch-duct IPMN with mural nodule (**a**) B-mode EUS; (**b**) CH-EUS-FNA from the mural nodule (arrow).

The septations hyperenhancement was noted in 12 IPMNs, seven SCNs, and three MCNs. Of 21 patients with a solid component on B-mode EUS, 13 patients had arterial enhancement, considered as mural nodules, with variable behavior of wash-out phase while the rest were mucus clots or debris (Figure 4 and Figure 5) (Supplementary Material—Table S2).

From the 13 mural nodules, the hypoenhancement was noted in two ductal cystic adenocarcinomas, one iso-enhancement was seen in one IPMN with confirmed malignancy, and the rest of the nodules were hyperenhanced in the arterial phase.

Different patterns of arterial enhancement and wash-out were observed according to mucinous vs. non-mucinous cysts and respectively malignant vs. non-malignant cysts. The arterial enhancement of the wall was more often seen in mucinous lesions than in non-mucinous lesions (*p* < 0.0001), with 90.2% sensitivity and 70,4% specificity, and good clinical utility index (0.784), but without importance for differentiating malignancy. The fast wash out of the wall was more frequently seen in mucinous (*p* < 0.0006) and malignant lesions (*p* < 0.0003), with good specificity (94.1% and 79,4%), but with low sensitivity (48% and 58.3%), and poor clinical utility index. The enhancement pattern in the septations was not helpful for differentiating cysts. (Table 2 and Table 3).

### 3.4. Contrast- EUS-FNA Assessment of Pancreatic Cysts 

The CH-EUS-FNA was performed in 48 of 58 cases (82.75%), 41 (87.5%) had conclusive results and it was considered unnecessary for typical lesions (Table 4, Supplementary Material—Table S3).

The CH-EUS-FNA diagnostic rate for mucinous cysts had a sensitivity of 82.4% and a specificity of 92.9% while for malignant cysts the sensitivity was 84.2% and a specificity of 100% (Table 4).

The fluid sample was enough for CEA determination in 28 patients. The CEA level was above the cutoff values in 9/28 cases, one case of ductal adenocarcinoma, four cases of MCN, cases and four IPMN cases.

The CH-EUS-FNA from mural nodule was performed in 13 patients, all had conclusive cytology, with low/moderate dysplasia in three cases (23.07%) (1 patient died non-related to the pancreatic lesion and two patients received surgical treatment during the follow-up) and high dysplasia/carcinoma in 10 (76.9%) (confirmed by surgery—6, unfit for surgery—4) (Table 4). There was one adverse event of mild acute pancreatitis with a good outcome (2%).

## 4. Discussion

This is the first study showing that contrast-enhanced guidance of FNA through an enhanced mural nodule in PCN was conclusive and positive for dysplasia/malignancy in 100% of cases and high-grade dysplasia or malignancy in 76.9%. Most of the nodules had no power Doppler signal. 

Also, we found that the arterial enhancement of the cystic wall was more frequently seen in mucinous lesions than non-mucinous PCN (Table 3), with a sensitivity of 90.2% and specificity of 70.6%, but without value for differentiation malignant PCN (Table 2), similar to other studies [6,7]. This feature was previously reported as being present in 89–90% of MCN but in 85–86% of SCN which is higher than we found (50%) [5,10].

The fast wash-out defined as starting before 45 s from the injection of the contrast was studied in our work. Starting from the presumption that liver malignant tumors provide a fast wash-out [10,20], a similar assessment could be designed for pancreatic cysts. However, the positive likelihood ratio for the fast wash-out of the cyst wall had modest values for mucinous cysts. The fast wash-out in our study was found in 37% of SCN and 75% of MCN, comparable to that reported in the literature of 22% and 13–89% [5]. This parameter proved better value for the malignant cysts, but with low sensitivity (Table 3), so we cannot rely on it for avoiding EUS-FNA. 

The presence of enhanced solid components over 5 mm represents an absolute indication for surgery [10,21] while the American guideline recommends resection of cysts with mural nodules, regardless of size [14]. CH-EUS can differentiate the unenhanced mucus or debris from malignant nodules (with dysplasia or invasive cancer) of MCN or IPMN, which are enhanced, with fast wash-out [5,6,7,22]. The presence of malignancy in mural nodules is detected in 88.9% of cases using Levovist [23], 70–100% using Sonazoid [5,22,24], and 76.9% in our study using Sonovue. CH-EUS detection rate of mural nodules was superior to standard EUS and CT scans in five studies using Sonazoid [9]. In our group, from 21 PCN with solid components (36%), only four had a power Doppler signal (6.8%), while 13 (22.4%) had an enhancing pattern on CH-EUS, being true mural nodules, so the use of contrast could avoid unnecessary EUS-FNA. 

Data from the literature shows that EUS-FNA of pancreatic cysts had a sensitivity of 54% for the discrimination of mucinous versus non-mucinous cysts [25], which increased from 44% to 78% when more passes from the solid component were targeted [26]. The malignancy diagnosis from the cystic fluid analysis was found as 32–58.6% [27,28] and we obtained 84.2% sensitivity when the CH-EUS-FNA from the fluid analysis and mural nodules were taken together, proving that the guiding EUS-FNA towards the enhanced solid intracystic component or hyperenhanced thick septation increases the number of conclusive pathological results, as suggested previously [6]. Identification of low/moderate dysplasia in 23% of mural nodules is important for close follow-up of patients with PCN. 

The use of through-the-needle biopsy (TTNB) was previously assessed and showed 83.7–88.6% sensitivity and 81.8–94.7% specificity for mucinous PCN [29,30], which are close to our results, but the rate of adverse events was 8.6–9.9% [29,30], which is higher than our rate of 2%. This raises the question of preferring the combination of EUS-FNA and contrast in PCN, but further research is needed [29,30].

The main limitation of our study is the lack of surgical pathology evaluation for all cases, so the standard reference was used as the surgical pathology or EUS-FNA cytology or on the correlation of clinical history, CT/MRI, B-mode EUS, and CH-EUS morphology, cyst fluid analysis and follow-up (clinical evaluation, serum CA19-9, abdominal ultrasound every six months and EUS or MRI every year for patients without surgical indication). Another limitation was the follow-up period of 25 months in case of no surgery or patient unfit for surgery (six patients) which limited determining the specific diagnosis in some cases which were excluded from the analysis. Additionally, no quantitative assessment of contrast images that might add supplementary data for differentiation of high-grade dysplasia/invasive carcinoma [31] was performed. The qualitative CH-EUS assessment used in this work is associated with a moderate interobserver agreement for the enhancement and fair for the wash-out) [32]. The use of EUS-FNB in the case of solid pancreatic masses proved to have better results than EUS-FNA [33], but their use in the case of PCN can be associated with a higher bleeding rate of up to 8.2% [34]. At the time of starting this study, anti-biotherapy prophylaxis was indicated, but this attitude is obsolete now [35].

In conclusion, the use of CH-EUS allowed the detection of the true mural nodules with enhanced appearance and guide EUS-FNA for detecting high-grade dysplasia/malignancy with 100% sensitivity. The cyst wall enhancement was useful to differentiate mucinous from non-mucinous cysts. The wash-out of contrast could discriminate between malignant and non-malignant PCN with modest accuracy. 

## Figures and Tables

**Table 1:** Patients’ demographics and tumor characteristics.

Parameters	Value
Age, years (mean ± SD)	59.8 ± 13.4
Sex female, *n* (%)	38 (65.5)
BMI, kg/m^2^ (mean ± SD)	26.1 ± 3.5
Size, mm Median (Q1 to Q3)	25 (18.3 to 40.0)
Number of lesions, *n* (%) Unifocal Multifocal	54 (93.1) 4 (6.9)
Location, *n* (%) Head + isthmus Body Tail Multiple	33 (56.9) 15 (25.9) 7 (12.1) 3 (5.2)
Final diagnosis, *n* (%) IPMN PK SCN Cystic ductal adk MCN Cystic acinar cell carcinoma	30 (51.7) 9 (15.5) 8 (13.8) 6 (10.3) 4 (6.9) 1 (1.7)

SD = standard deviation, SCN- serous cystic neoplasms, MCN—mucinous cystic neoplasms, BD-IPMN—branch duct intrapapillary mucinous neoplasm, PK—pseudocyst, adk—adenocarcinoma.

**Table 2:** CH-EUS features: mucinous vs. non-mucinous and malignant vs. non-malignant pancreatic cysts.

Cyst Feature	Mucinous Cyst			Malignant Cyst		
	Yes (*n* = 41)	No (*n* = 17)	*p*-Value	Yes (*n* = 19)	No (*n* = 39)	*p*-Value
***Wall***						
Arterial enhancement/*n* (%) Fast venous wash-out/*n* (%)	37/41 (90.2) 20/41 (48.78)	5/17 (29.41) 1/17 (5.88)	<0.0001 0.0006	15/19 (78.94) 11/19 (57.89)	27/39 (69.23) 10/39 (25.64)	0.4371 0.0164
Septation *						
Arterial enhancement/*n* (%) Fast venous wash-out/*n* (%)	17/29 (58.62) 10/29 (34.48)	7/10 (70) 2/10 (20)	0.9839 0.3923	8/13 (61.53) 5/13 (38.46)	16/26 (61.53) 7/26 (26.92)	>0.9999 0.3776
***Mural nodule***						
Arterial enhancement/*n* (%) Fast venous wash-out/*n* (%)	13/13 (100) 8/13 (61.53)	0/0 0/0	n.a. n.a.	10/10 (100) 7/10 (70)	3/3 (100) 1/3 (30)	n.a. 0.6294

Data are reported as numbers; *p*-value reflects the Chi-squared or Fisher’s exact test; n.a. = not appropriate; *—only a part of patients with cysts presented septation.

**Table 3:** CH-EUS performances as a diagnosis tool.

	Arterial Enhancement of the Wall for Diagnosing Mucinous Cyst	Arterial Enhancement of the Mural Nodules in Diagnosing Malignant Cyst	Fast Venous Wash Out of the Wall for Diagnosing Mucinous Cysts	Fast Venous Wash Out of the Wall for Diagnosing Malignant Cyst
Se% [95%CI]	90.2 [81.2 to 99.3]	100	48.8 [33.5 to 64.1]	58.3 [38.6 to 78.1]
Sp% [95%CI]	70.6 [48.9 to 92.2]	n.a.	94.1 [82.9 to 100]	79.4 [65.8 to 93.0]
Acc% [95%CI]	84.5 [75.2 to 93.8]	76.9 [54.0 to 99.8]	62.07 [49.6 to 74.6]	70.7 [59.0 to 82.4]
PPV% [95%CI]	88.1 [78.3 to 97.9]	76.9 [54.0 to 99.8]	95.2 [86.1 to 100]	66.7 [46.5 to 86.8]
NPV% [95%CI]	75.0 [53.8 to 96.2]	n.a.	43.2 [27.3 to 59.2]	73.0 [58.7 to 87.3]
+LR [95%CI]	3.07 [1.46 to 6.45]	n.a.	8.29 [1.21 to 56.8]	2.83 [1.35 to 5.95]
−LR [95%CI]	0.14 [0.05 to 0.37]	n.a.	0.54 [0.39 to 0.75]	0.52 [0.32 to 0.87]
+CUI [95%CI]	0.795 [0.679 to 0.911]	0.769 [0.543 to 0.995]	0.465 [0.271 to 0.658]	0.389 [0.144 to 0.634]
−CUI [95%CI]	0.529 [0.342 to 0.717]	n.a.	0.407 [0.268 to 0.546]	0.579 [0.459 to 0.700]

Se = sensitivity; Sp = specificity; Acc = accuracy; PPV = positive predictive value; NPV = negative predictive value; LR = Likelihood Ratio; CUI = clinical utility index; + = positive; − = negative.

**Table 4:** Contrast-EUS-FNA cytology assessment of pancreatic cysts.

Diagnosis of Cysts Assessed with CH- EUS-FNA	Mucinous	Non-Mucinous	Malignant	Non-Malignant
**Fluid + mural nodules cytology (*n* = 48)**	34	14	16	25
Conclusive (*n* = 41)	27	14	16	18
High dysplasia/carcinoma * Low/moderate dysplasia * No dysplasia *	16 5 6	0 0 14	16 0 0	0 5 6
**Mural nodule cytology (*n* = 13)**	13	0	10	3
Conclusive (*n* = 13)	13	0	10	3
High dysplasia/carcinoma * Low/moderate dysplasia *	10 3	0 0	0 0	0 0
Diagnostic rate	Mucinous vs. Non-mucinous		Malignant vs. Non-malignant	
Sensitivity (%) Specificity (%) Positive predictive value Negative predictive value +CUI [95%CI] −CUI [95%CI]	82.4 92.9 96.6 68.4 0.795 [0.66 to 0.92] 0.635 [0.47 to 0.79]		84.2 100 100 90.6 0.842 [0.68 to 0.997] 0.906 [0.84 to 0.97]	

Data are expressed as absolute frequency; * data are expressed only for conclusive results; SCN—serous cystic neoplasms, PK—pseudocyst, BD-IPMN- branch duct intrapapillary mucinous neoplasm, MCN—mucinous cystic neoplasms, adk—adenocarcinoma.

## Data Availability

Raw data and study materials that support the findings are available to other researchers from the first author (M.P.O.) upon request.

## Supplementary Materials

The following supporting information can be downloaded at: https://www.mdpi.com/article/10.3390/diagnostics12092209/s1, Table S1: B-mode EUS aspects according to the final diagnosis; Table S2: Arterial enhancement and venous wash-out on the wall, septa, and solid component by type of pancreatic cysts; Table S3: Contrast-EUS-FNA cytology assessment of specific pancreatic cysts.
