# Supplementary material for: Clinical Utility of the Contrast-Enhanced Endoscopic Ultrasound Guided Fine Needle Aspiration in the Diagnosis of Pancreatic Cyst
Source: Diagnostics (Basel). 2022 Sep 12;12(9):2209. doi: 10.3390/diagnostics12092209 (PMC9497576; doi:10.3390/diagnostics12092209)
Supplement: Supplementary file 1 [file diagnostics-12-02209-s001.zip › diagnostics-1904916-supplementary.pdf]

Article

Clinical Utility of the Contrast-Enhanced Endoscopic Ultrasound Guided Fine Needle Aspiration in the Diagnosis of Pancreatic Cyst

Miruna Patricia Olar [1], Sorana D. Bolboacă [2,*], Cristina Pojoga [1,3,4], Ofelia Moșteanu [1,3], Marcel Gheorghiu [1,3], Radu Seicean [5], Ioana Rusu [1,3], Zeno Sparchez [1,3], Nadim Al Hajjar [3,6] and Andrada Seicean [1,3]

Supplementary Materials

Table S1. B-mode EUS aspects according to the final diagnosis.

Cyst features	IPMN (n=30)	PK (n=9)	SCN (n=8)	Cystic Ductal adk (n=6)	MCN (n=4)	Cystic acinar cell carcinoma (n=1)	Total (n=58)
Communication with MPD	9	2	0	0	0	0	11
MPD size							
5-9 mm	4	0	1	1	0	0	6
≥10 mm	2	0	0	0	0	0	2
Mural nodules	7	0	0	5	0	1	13
Solid mass	10	2	0	6	2	1	21
Septae	20	2	8	5	3	1	31
Septae>2 mm	3	0	0	3	0	1	7

Data are expressed as numbers; IPMN- branch duct intrapapillary mucinous neoplasm, PK- pseudocyst, SCN- serous cystic neoplasms, MCN- mucinous cystic neoplasms, MPD- main pancreatic duct

Table S2. Arterial enhancement and venous wash-out on the wall, septa and solid component by type of pancreatic cysts.

CH-EUS features	SCN (n=8)	PK (n=9)	IPMN (n=30)	MCN (n=4)	Cystic acinar cell carcinoma (n=1)	Cystic ductal adk (n=6)
			Wall			
Arterial enhancement						
yes	4	1	30	4	1	6
no	4	8	0	0	0	0
Venous wash-out						
fast	1	0	13	3	1	3
slow	3	3	17	1	0	0
			Septae			
Arterial enhancement						
yes	7	0	12	3	1	3
no	0	0	0	0	0	0
Venous wash-out						
fast	2	0	6	2	1	1
slow	5	0	6	1	0	2
Solid component	0	2	10	2	1	6
Mural nodule	0	0	7	0	1	5
Arterial enhancement						
hyper	0	0	6	0	1	3
hypo	0	0	0	0	0	2
iso	0	0	1	0	0	
Venous wash-out						
fast	0	0	4	0	1	3
slow	0	0	3	0	0	2

Data are expressed as numbers; SCN- serous cystic neoplasms, PK- pseudocyst, IPMN- branch duct intrapapillary mucinous neoplasm, MCN- mucinous cystic neoplasms, adk- adenocarcinoma, MPD- main pancreatic duct

Table S3. Contrast-EUS-FNA cytology assessment of specific pancreatic cysts.

Final diagnosis of cysts assessed With CH- EUS-FNA	SCN (n=7)	PK (n=7)	IPMN (n=23)	MCN (n=4)	Cystic acinar cell carcinoma (n=1)	Cystic ductal adk (n=6)
**Fluid + mural noduls cytology (n=48)**						
Conclusive/inconclusive (n=41/7)	7/0	7/0	19/4	2/2	1/0	5/1
Malignant/non-malignant (16/25)	0/7	0/7	9/9	2/2	1/0	5/0
High dysplasia / carcinoma	-	-	9/19	1/2	1/0	0/5
Low / moderate dysplasia	-	-	4/19	1/2	-	-
No dysplasia	0/7	0/7	6/19	-	-	-
**Mural nodule cytology (n=13)**						
Conclusive/inclonclusive (13/0)	0/0	0/0	7/0	0/0	1/0	5/0
Malignant/non-malignant (10/3)	0/0	0/0	4/3	0/0	1/0	5/0
High dysplasia / carcinoma (n=10)	-	-	4	-	1	5
Low / moderate dysplasia (n=3)	-	-	3	0	-	0

SCN- serous cystic neoplasms, PK- pseudocyst, IPMN- branch duct intrapapillary mucinous neoplasm, MCN- mucinous cystic neoplasms, adk- adenocarcinoma
